# miR-338-3p inhibits cell growth, invasion, and EMT process in neuroblastoma through targeting MMP-2

**DOI:** 10.1515/biol-2021-0013

**Published:** 2021-03-05

**Authors:** Haibin Yuan, Fengli Liu, Tongsheng Ma, Zhandong Zeng, Ning Zhang

**Affiliations:** Department of Neonatal Surgery, Xuzhou Children’s Hospital, No.18 Sudi North Road, Quanshan District, 221001, Xuzhou, China

**Keywords:** neuroblastoma, miR-338-3p, MMP-2, EMT pathway, PI3K/AKT signaling

## Abstract

This study aimed to explore the regulatory mechanisms of miR-338-3p and matrix metalloproteinase-2 (MMP-2) in neuroblastoma. Putative target interaction regions of miR-338-3p on MMP-2 were predicted by miRcode and miRbase bioinformatics tools. Relative expression of miRNA-338-3p and MMP-2 in neuroblastoma tissues and GI-LI-N and SK-N-SH cells was determined by reverse transcription polymerase chain reaction experiment. Furthermore, the cell proliferation was determined by Cell Counting Kit-8 assay, the cell apoptosis rate was analyzed by flow cytometry assay, and the cell invasion was evaluated by transwell assay. miR-338-3p expression was downregulated, whereas MMP-2 expression was upregulated in metastasis tissue site compared to that in primary tissue site in total. Furthermore, miR-338-3p overexpression suppressed proliferation, invasion, and epithelial–mesenchymal transition (EMT) of neuroblastoma cells but promoted apoptosis, and the knockdown of MMP-2 triggered similar effects. Furthermore, MMP-2 was directly targeted by miR-338-3p, and overexpression of MMP-2 rescued the inhibitory effects of miR-338-3p on human neuroblastoma cell progression. Collectively, these data demonstrated that miR-338-3p could suppress cell growth, invasion, and EMT pathway and induce apoptosis in neuroblastoma cells by targeting MMP-2. MiR-338-3p sponged MMP-2 to regulate the PI3K/AKT pathway in human neuroblastoma cells.

## 1 Introduction

As the most common extracranial solid tumor in infancy, neuroblastoma is derived from cells of neural crest origin and is notable for its broad range of clinical behaviors [1,2,3]. Neuroblastoma, with a survival rate of 40–50%, is continually unresectable or metastatic in the children aged 18 months or older, requiring intensive multimodality therapy [4].

MicroRNAs (miRNAs) are single-stranded non-coding RNA molecules containing about 22 nucleotides, which are involved in post-transcriptional regulation of gene expression [5]. In recent years, it has been reported that miRNAs may function as tumor suppressors, oncogenes, or candidate biomarkers in various cancers, including neuroblastoma, by regulating proliferation, angiogenesis, invasion, metastasis, and apoptosis [6]. For example, miR-205, miR-335, miR-363, and miR-542-3p were confirmed to have an inhibitive effect on neuroblastoma tumorigenesis, growth, and metastasis [7,8,9], whereas miRNA-21 and miRNA-558 were ascertained with a promotive effect on neuroblastoma tumorigenesis, proliferation, and invasion [10,11]. Moreover, it was reported that miRNA-221 as an oncogene was related to poor prognosis in neuroblastoma [12]. Importantly, it was found that miR-338-3p was downregulated and - through the specific targets - suppressed the cell growth and invasion in various cancers, such as ovarian cancer, gastric cancer, breast tumor, prostate cancer, non-small cell lung cancer, glioblastoma, and neuroblastoma [13,14,15,16,17,18,19,20].

Known as inflammatory mediators, matrix metalloproteinases (MMPs) are a family of zinc-dependent endopeptidases involved in physiological and pathological processes [21]. MMPs are responsible for cleaving protein substrates, and their major functions are degradation of extracellular matrix to facilitate the progression of cell migration and invasion, regulation of vascular cell proliferation and apoptosis via regulating protein cleavage, and modulation of bioactive molecules and relevant signaling pathways [22].

However, there was no study exploring the target relationship and regulatory mechanism between miR-338-3p and matrix metalloproteinase-2 (MMP-2) in neuroblastoma. Considering the miR-338-3p putative target interaction region in MMP-2, predicted by miRcode and miRbase bioinformatics tools, we hypothesized that miR-338-3p might inhibit the cell invasion and epithelial–mesenchymal transition (EMT) process in neuroblastoma by targeting MMP-2, and the hypothesis was tested in GI-LI-N and SK-N-SH neuroblastoma cell lines. Overall, those research findings confirmed the hypothesis that miR-338-3p could inhibit the cell invasion and EMT process in neuroblastoma by targeting MMP-2.

## 2 Materials and methods

### 2.1 Tissue samples

As the experimental samples, 20 pairs of human metastasis and primary tissues from neuroblastoma patients were obtained from the Xuzhou Children’s Hospital of Medical College between March 2017 and August 2019.


**Informed consent:** Informed consent was obtained from all the individuals included in this study.
**Ethical approval:** The research related to human use has been complied with all the relevant national regulations and institutional policies and in accordance with the tenets of the Helsinki Declaration and has been approved by the Ethics Committee of Xuzhou Children’s Hospital, China.

### 2.2 Cell culture

Human neuroblastoma cell lines GI-LI-N and SK-N-SH were purchased from Procell Life Science and Technology (Wuhan, China) and cultured in Dulbecco’s modified Eagle medium (DMEM) supplemented with 10% fetal bovine serum (FBS) and 2 mmol/L l-glutamine (HyClone, Logan, UT, USA). All cells were incubated at 37°C, 5% CO_2_ humidified atmosphere.

### 2.3 Transfection

The overexpression and knockdown vectors of miR-338-3p and MMP-2 were purchased from Shanghai GenePharma (Shanghai, China) named as miR-338-3p mimics, miR-338-3p inhibitor, pc-MMP-2, and si-MMP-2, which were in comparison with negative control (NC) named as NC mimic, NC inhibitor, pc-NC, and si-NC, respectively. When the cells’ culture confluency reached 50%, the overexpression and knockdown vectors of miR-338-3p, MMP-2, and NC were transfected into the cells with Lipofectamine-2000 (Invitrogen, Carlsbad, CA, USA) following the protocol of the manufacturer. Transfected cells were harvested for 24, 48, or 72 h for further experiments.

### 2.4 RNA extraction and reverse transcription polymerase chain reaction (RT-PCR)

Total RNA of human neuroblastoma tissues and cell lines was extracted by Trizol reagent (Invitrogen), and the reverse transcription was applied by the PrimeScript RT Reagent kit (Takara Bio, Inc., Otsu, Japan). RT-PCR was conducted with SYBR Premix Ex Taq II reagent by 7500 RT-PCR System (Applied Biosystems, Foster City, CA, USA). For miR-338-3p, RT-PCR was conducted with PrimeScript^®^ miRNA RT-PCR kit (Takara) according to the manufacturer’s instructions. In addition, the primers for RT-PCR were purchased from TIANGEN (Shanghai, China). Relative expression level of miR-338-3p was normalized to the internal control U6 snRNA while relative expression level of MMP-2 was normalized with internal control GAPDH, and were calculated by the 2^−∆∆Ct^ method. The sequences of the primers in this study are presented in Table 1.

**Table 1:** Primer sequence information for qRT-PCR

Gene	Primer	*T*_m_	Fragment size	Amplification efficiency (%)
MMP-2	5′-GGCCCTGTCACTCCTGAGAT-3′ (forward)	60.98	474	95.42
	5′-GGCATCCAGGTTATCGGGGA-3′ (reverse)	61.42		
GAPDH	5′-GGGTGATGCAGGTGCTACTT-3′ (forward)	60.04	499	98.25
	5′-GGCAGGTTTCTCAAGACGGA-3′ (reverse)	59.97		
miR-338-3p	5′-TGCGGTCCAGCATCAGTGAT-3′ (forward)	61.90	22	96.46
	5′-CCAGTGCAGGGTCCGAGGT-3′ (reverse)	63.86		
U6	5′-GCTCGCTTCGGCAGCACA-3′ (forward)	63.70	107	96.77
	5′-GAGGTATTCGCACCAGAGGA-3′ (reverse)	58.89		

### 2.5 Cell proliferation assay

To explore the cell proliferation, Cell Counting Kit-8 (CCK-8; Tianjin Biolite, Tianjin, China) assay was adopted. After being cultured for 24 h, GI-LI-N and SK-N-SH cells transfected with miR-338-3p mimics, miR-338-3p inhibitor, pc-MMP-2, and si-MMP-2, or corresponding NC vectors were plated into 96-well plates (Corning Costar, Corning, NY, USA) at a density of 2.0 × 10^3^ cells per well. The cell proliferation was analyzed at 24, 48, and 72 h post-transfection. The absorbance was measured at 450 nm by one microplate reader (Thermo Fisher Scientific, Waltham, MA, USA).

### 2.6 Flow cytometry assay

Transfected GI-LI-N and SK-N-SH cells were collected and washed with phosphate-buffered saline (PBS). Annexin V-fluorescein isothiocyanate Apoptosis Detection kit (Thermo Fisher Scientific) was applied for apoptosis detection, and then the cell apoptosis rate was tested by NovoCyte Flow Cytometer (ACEA Biosciences Inc., San Diego, CA, USA). Every assay was conducted in triplicate.

### 2.7 Transwell invasion assay

Transwell assay was used to examine the cell invasion capability of GI-LI-N and SK-N-SH cells. Cells were transfected with miR-338-3p mimic, si-MMP-2, pc-MMP-2, and corresponding NC vectors. After 24 h, transfected cells were harvested, and 1.0 × 10^4^ cells in 200 μL of DMEM were placed into the upper chambers, which were pre-coated with Matrigel (BD Biosciences, San Jose, CA, USA). Then the lower chambers were filled with 600 μL of DMEM with 10% FBS. After 12 h of incubation with 5% CO_2_ at 37°C, the fraction of non-invading cells was removed from the top of the chamber with cotton swabs. Furthermore, the invasion cells on the lower surface of the inserts were stained with 0.1% crystal violet and analyzed with the microscope.

### 2.8 Western blot assay

GI-LI-N and SK-N-SH cells were harvested and lysed with RIPA cell lysis buffer (Thermo Fisher Scientific) with protease inhibitors. The protein concentration was measured by bicinchoninic acid method (Thermo Fisher Scientific). Then the extracted protein samples were separated by 10% sodium dodecyl sulfate-polyacrylamide gel electrophoresis and transferred onto the polyvinylidene difluoride (PVDF) membrane (Millipore, Bedford, MA, USA) on ice. Next, after being blocked for 1 h with 5% non-fat milk at room temperature, the PVDF membranes were incubated with primary antibodies overnight at 4°C. MMP-2 antibody (1:2,000), E-cadherin antibody (1:2,000), N-cadherin antibody (1:1,000), vimentin antibody (1:1,000), phosphatidylinositol 3-kinase (PI3K) (#4,257, 1:1,000), phosphorylated (p)-PI3K (#17,366, 1:1,000), serine/threonine kinase 1 (AKT) (#9,272, 1:1,000), p-AKT (#9,611, 1:1,000), and GAPDH antibody (1:1,000) were purchased from Affinity Biosciences (Changzhou, China). Moreover, membranes were incubated with the corresponding HRP-conjugated second antibodies HRP Goat Anti-Rabbit IgG (H+L) (1: 5000, ABclonal, Wuhan, china) or HRP Goat Anti-Mouse IgG (H+L) (1: 5000, ABclonal), and protein signals were visualized with enhanced chemiluminescence (Millipore).

### 2.9 Dual-luciferase reporter assay

For the putative binding sequence of miR-338-3p on MMP-2 predicted by online bioinformatics databases, the MMP-2 putative binding sequence was cloned into the downstream of firefly luciferase gene of pGL3 luciferase promoter vector (Promega Corporation, Madison, WI, USA), named as WT MMP-2. In addition, the mutant potential binding site was cloned into the pGL3 vector, named as MUT MMP-2. GI-LI-N and SK-N-SH cells were placed into the 24-well plate for 48 h after co-transfection with 100 ng WT MMP-2 or MUT MMP-2, 50 nM miR-338-3p or miR-NC, and 50 nM of Renilla luciferase reporter vector (pRL-TK, Promega) using the Lipofectamine™ 3000 reagent (Life Technologies Corporation, Carlsbad, CA, USA). Furthermore, all the cells were collected and luciferase activity was determined by dual-luciferase reporter assay kit (Promega).

### 2.10 Statistical analysis

All data were presented as mean ± standard deviation, which was analyzed by GraphPad Prism 7 software (GraphPad Software, Inc. 7825 Fay Avenue, Suite 230 La Jolla, CA, USA). Difference analysis was performed by LSD-t (for more than three group data) and Student’s *t*-test (for two data). Rank correlation was also used. Each experiment was conducted in triplicate. Meanwhile, a significant difference was confirmed with a *P*-value of less than 0.05 in statistical analysis (*P* < 0.05).

## 3 Results

### 3.1 miR-338-3p expression in metastasis tissue site was downregulated compared to that in primary tissue site in total

**Figure 1:**
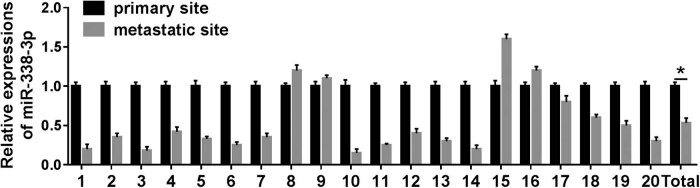
The expression of miR-338-3p in human neuroblastoma tissues. The differential expression of miR-338-3p in primary tissue site and metastasis tissue site in 20 pairs of neuroblastoma samples was detected by RT-PCR normalized to GAPDH. *P < 0.05.

To determine the latent effect of the miR-338-3p in the process of human neuroblastoma metastasis, the expression level of miR-338-3p in 20 pairs of human neuroblastoma tumor tissues was measured. The expression level of miR-338-3p was dramatically downregulated compared with that in the primary site in total (Figure 1). Interestingly, the miR-338-3p expression of neuroblastoma patients was associated with clinical features (Table 2).

**Table 2:** Relationship between miR-338-3p expression and clinical features of NB patients (*n* = 20)

Parameters	Case	miR-338-3p expression^a^		*P*-value
		High (*n* = 11)	Low (*n* = 9)	
**Age (years)**				
≤2.5	7	3	4	0.352
>2.5	13	8	5	
**Gender**				
Female	14	8	6	0.4212
Male	6	3	3	
**INSS stage**				
1–2	11	5	6	0.0132*
3–4S	9	6	3	
**Lymph node metastasis**				
No	11	5	6	0.0165*
Yes	9	6	3	

Note: *P < 0.05.

a Using median expression level of miR-338-3p as cutoff.

### 3.2 Overexpression of miR-338-3p suppressed the human neuroblastoma cell proliferation and invasion, whereas it promoted the cell apoptosis

**Figure 2:**
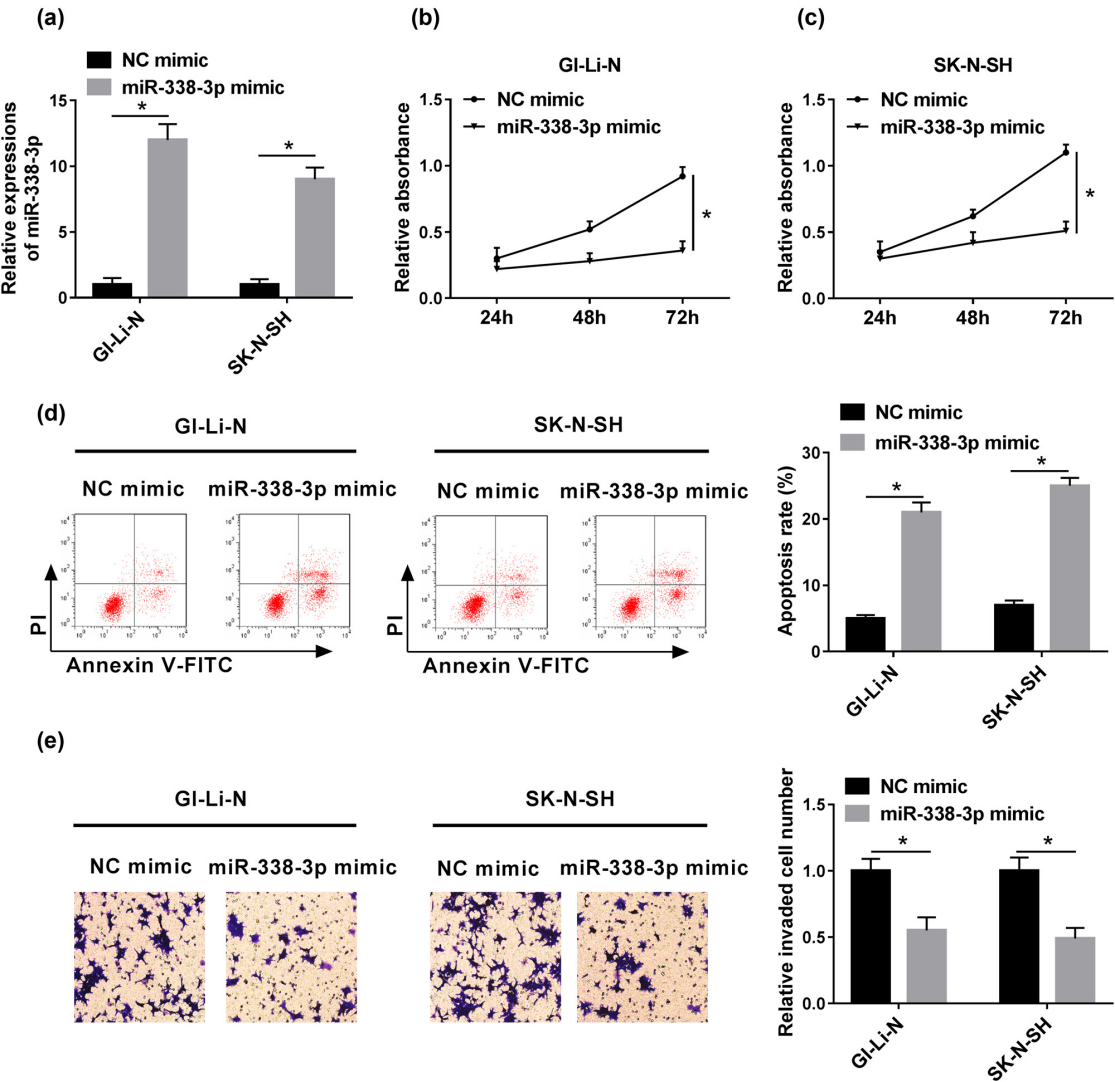
miR-338-3p mimic inhibited cell proliferation and invasion but promoted apoptosis *in vitro*. GI-LI-N and SK-N-SH cells were transfected with NC mimics or miR-338-3p mimics. (a) The expression of miR-338-3p was detected by RT-PCR after transfection. (b and c) CCK-8 assay at 450 nm was applied to examine cell proliferation of transfected cells. (d) The cell apoptosis was detected via flow cytometry assay. (e) The cell invasion was analyzed via transwell assay. *P < 0.05.

To further explore the effect of miR-338-3p in human neuroblastoma, miR-338-3p mimics were synthesized and transfected into GI-LI-N and SK-N-SH cell lines, separately. The expression of miR-338-3p in miR-338-3p mimic groups was dramatically enhanced in comparison with that in the NC mimic group (Figure 2a). Moreover, the cell proliferation in GI-LI-N and SK-N-SH cells with miR-338-3p mimics was apparently inhibited in comparison to those in NC mimic, whereas the cell apoptosis was remarkably intensified (Figure 2b–d). Furthermore, the cell invasion in miR-338-3p mimic groups was also conspicuously retarded (Figure 2e).

### 3.3 Overexpression of miR-338-3p repressed EMT in human neuroblastoma cells

**Figure 3:**
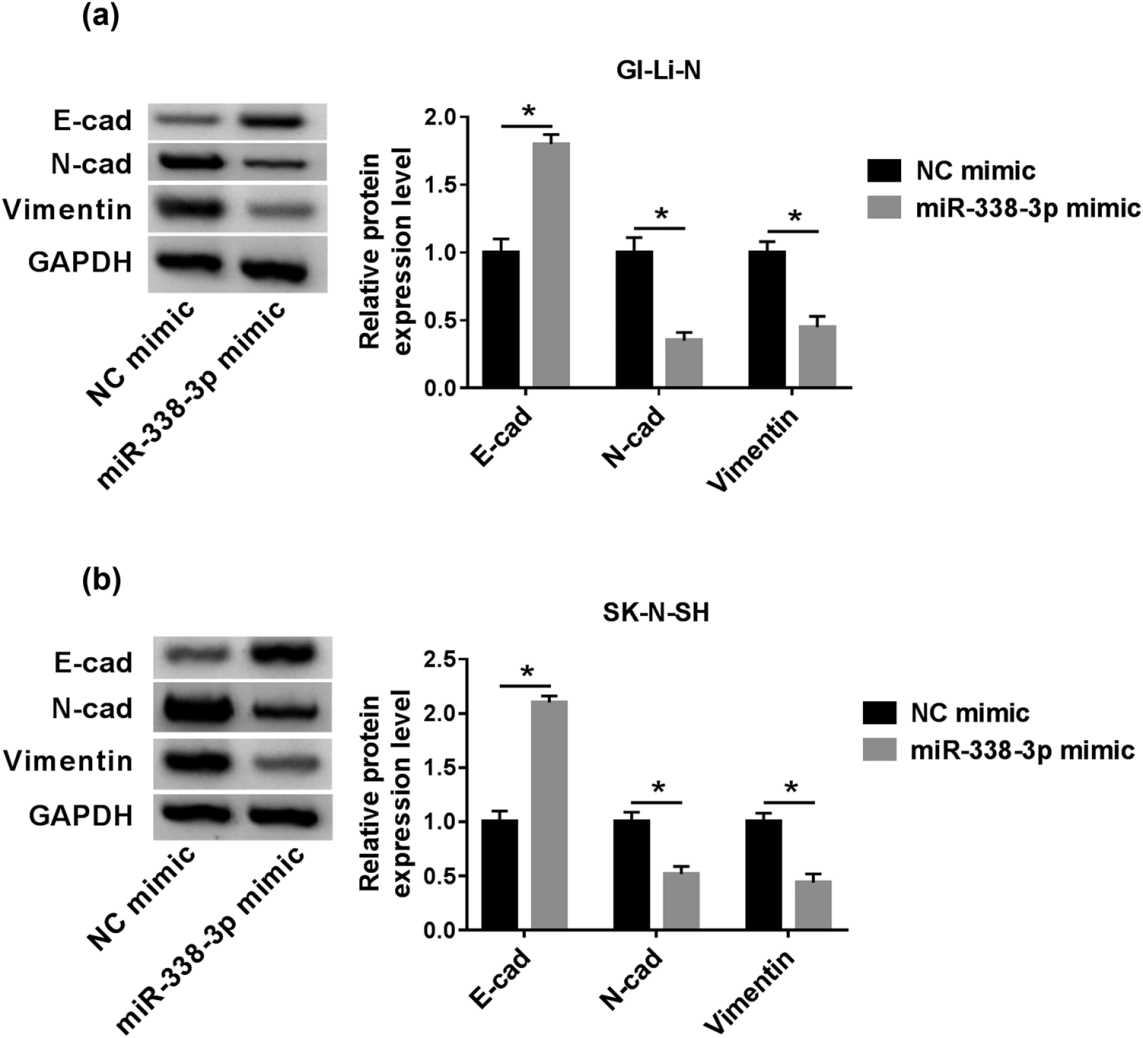
Upregulated miR-338-3p inhibited EMT progression *in vitro*. GI-LI-N and SK-N-SH cells were transfected with NC mimics or miR-338-3p mimics. (a and b) The expression of EMT-related proteins was examined by western blot assay. *P < 0.05.

E-cadherin, N-cadherin, and vimentin proteins were the three EMT process markers. E-cadherin protein expression in miR-338-3p mimic groups was enhanced compared with that in the NC mimic group, whereas N-cadherin and vimentin expression was prominently downregulated (Figure 3a and b). This result indicated that overexpression of miR-338-3p restrained the EMT pathway.

### 3.4 MMP-2 was directly targeted by miR-338-3p

**Figure 4:**
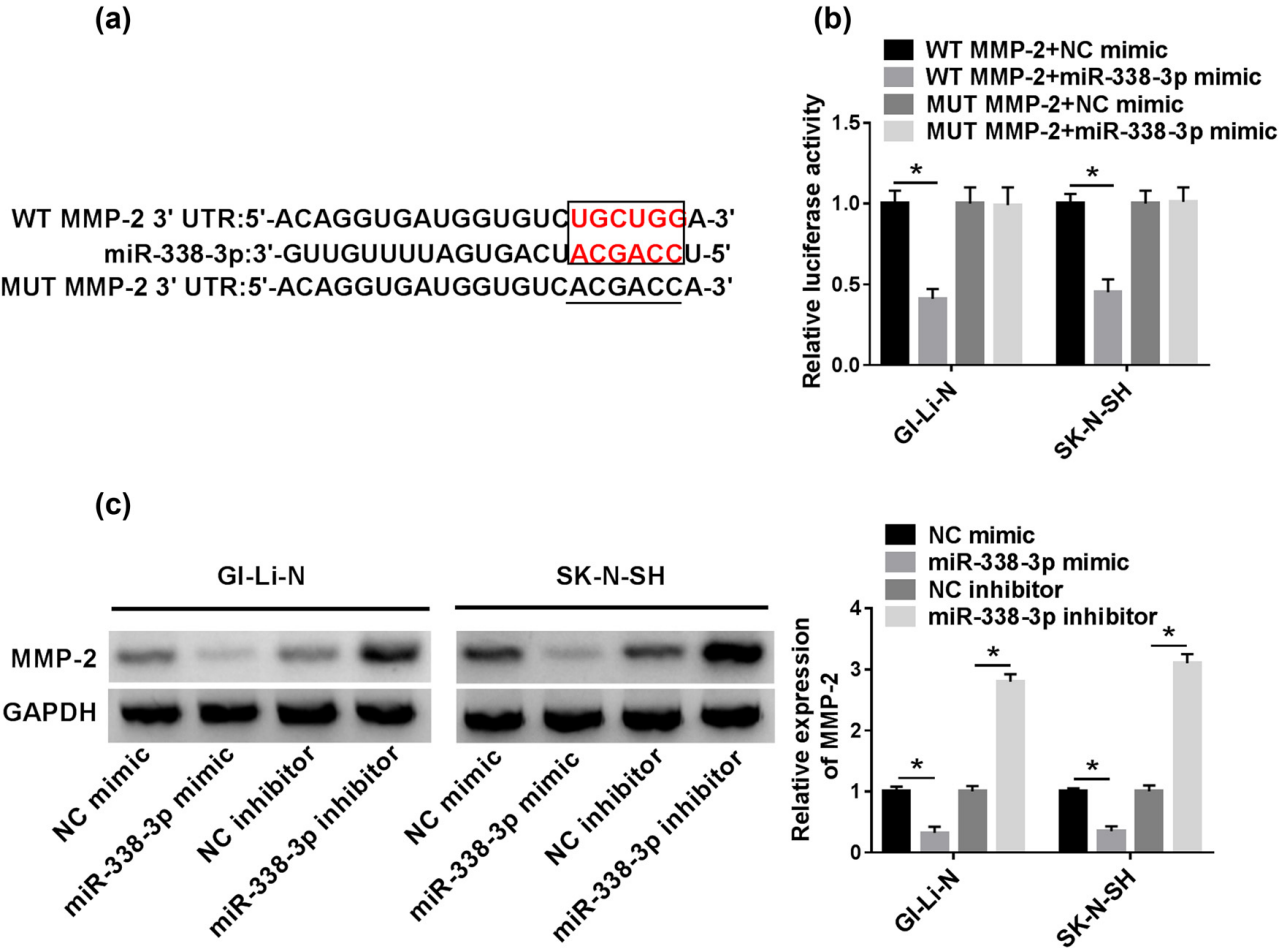
The relationship between MMP-2 and miR-338-3p. (a) Predicted binding site between miR-338-3p and MMP-2 was exhibited. (b) The luciferase activity was analyzed by dual-luciferase reporter assay. (c) MMP-2 expression was measured by western blot assay. *P < 0.05.

For further investigating the molecular mechanism of miR-338-3p in human neuroblastoma cell invasion, the potential binding site between miR-338-3p and MMP-2 was predicted through the miRcode and miRbase online bioinformatics tools (Figure 4a). Dual-luciferase and western blot assays were conducted to validate the relationship between miR-338-3p and MMP-2. The luciferase activity of GI-LI-N and SK-N-SH cells co-transfected with WT MMP-2 and miR-338-3p mimic was substantially constrained in comparison with that in cells co-transfected with WT MMP-2 and NC mimic. However, there was no significant difference in luciferase activity of cells transfected with MUT MMP-2 (Figure 4b). In addition, the MMP-2 protein expression in GI-LI-N and SK-N-SH cells transfected with miR-338-3p mimic was repressed relative to that in the NC mimic group, whereas the expression level of MMP-2 protein in cells transfected with miR-338-3p inhibitor was robustly increased compared to that transfected with NC inhibitor (Figure 4c). Therefore, this result presented the target relationship between miR-338-3p and MMP-2.

### 3.5 MMP-2 was upregulated in human neuroblastoma tissues

**Figure 5:**
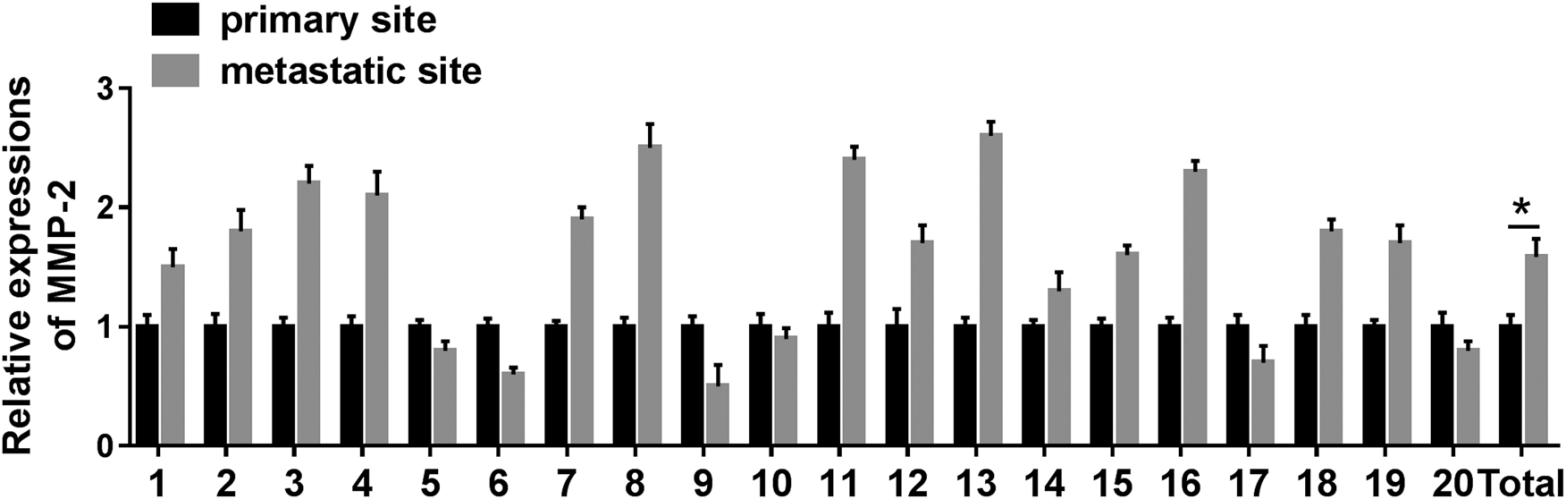
The expression of MMP-2 in 20 pairs of neuroblastoma samples. Expression of MMP-2 in metastasis tissue site and primary tissue site was measured by RT-PCR. *P < 0.05.

To disclose the effect of MMP-2 in human neuroblastoma, the expression level of MMP-2 of 20 pairs of human neuroblastoma tissues was measured by RT-PCR. The result indicated that MMP-2 expression in the metastatic site was exceptionally upregulated relative to that in the primary site in total (Figure 5).

### 3.6 Knockdown of MMP-2 inhibited neuroblastoma cell progression

**Figure 6:**
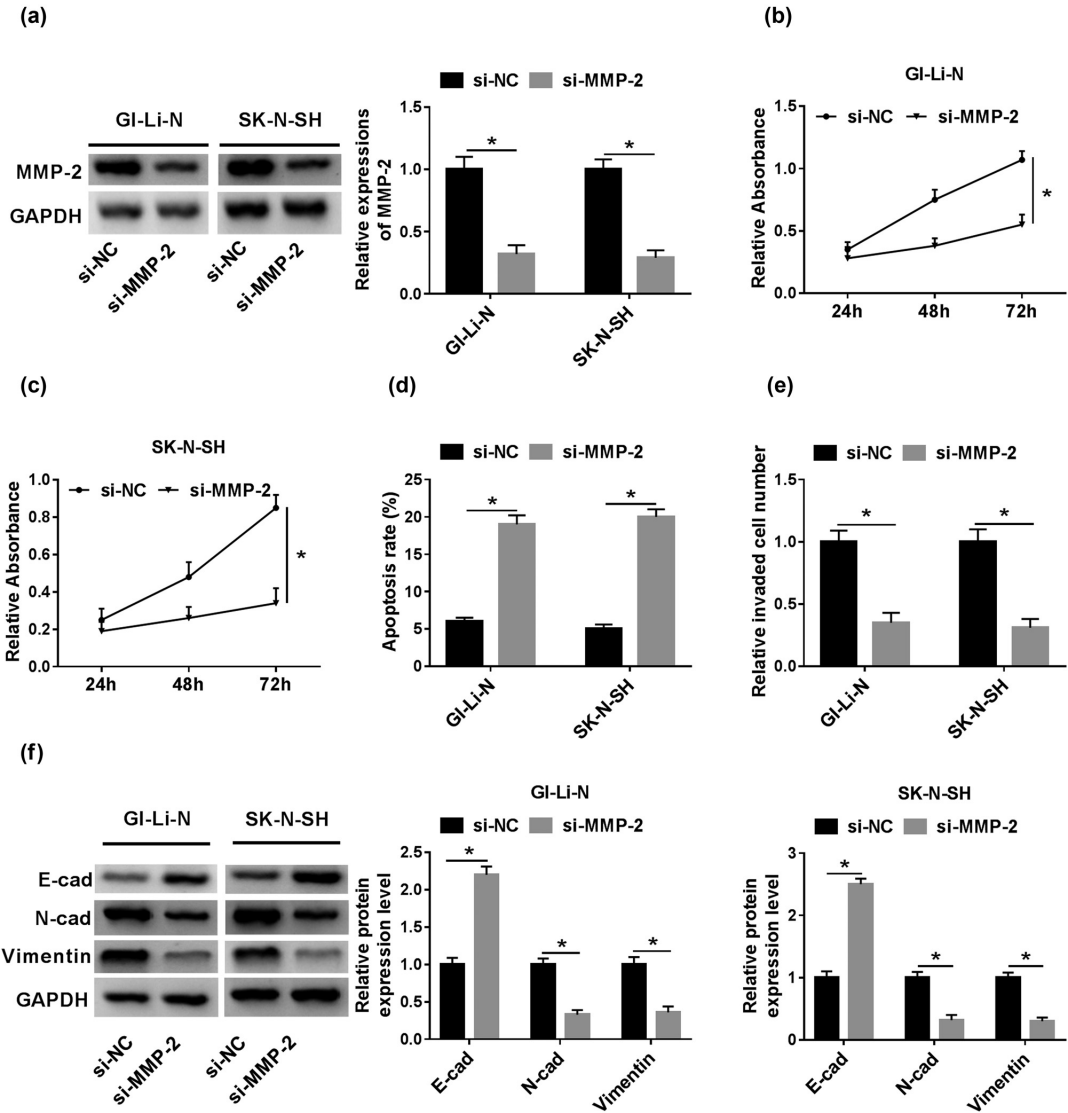
si-MMP-2 inhibited the cell proliferation, invasion, and EMT but promoted the cell apoptosis rate *in vitro*. (a) MMP-2 protein expression in GI-LI-N and SK-N-SH cells with si-MMP-2 was measured by western blot assay and normalized to GAPDH. (b and c) Cell proliferation of the cells with si-MMP-2 or si-NC was detected by CCK-8 assay at 450 nm. (d) Flow cytometry was used to analyze the cell apoptosis rate in cells with si-MMP-2 or si-NC. (e) Cell invasion in si-MMP-2 or si-NC groups was confirmed by transwell assay. (f) Western blot assay was adopted to visualize the E-cadherin, N-cadherin, and vimentin protein expression of cells with si-MMP-2 or si-NC. *P < 0.05.

To further illustrate the effect of MMP-2 in human neuroblastoma, si-MMP-2 vector was constructed to silence the MMP-2. MMP-2 protein expression of GI-LI-N and SK-N-SH cells transfected with si-MMP-2 was robustly decreased compared to that of cells transfected with si-NC (Figure 6a). Cell proliferation in GI-LI-N and SK-N-SH transfected cells with si-MMP-2 was strikingly constrained relative to that in cells with si-NC (Figure 6b and c). However, the cell apoptosis rate in GI-LI-N and SK-N-SH cells with si-MMP-2 was specially facilitated (Figure 6d). Furthermore, the cell invasion of transfected si-MMP-2 cells was prominently restrained in comparison with that in cells with si-NC (Figure 6e). Interestingly, the EMT process in si-MMP-2 groups was abnormally blocked in contrast to that in si-NC groups (Figure 6f).

### 3.7 MMP-2 rescued the inhibitory effects of miR-338-3p on human neuroblastoma cell progression

**Figure 7:**
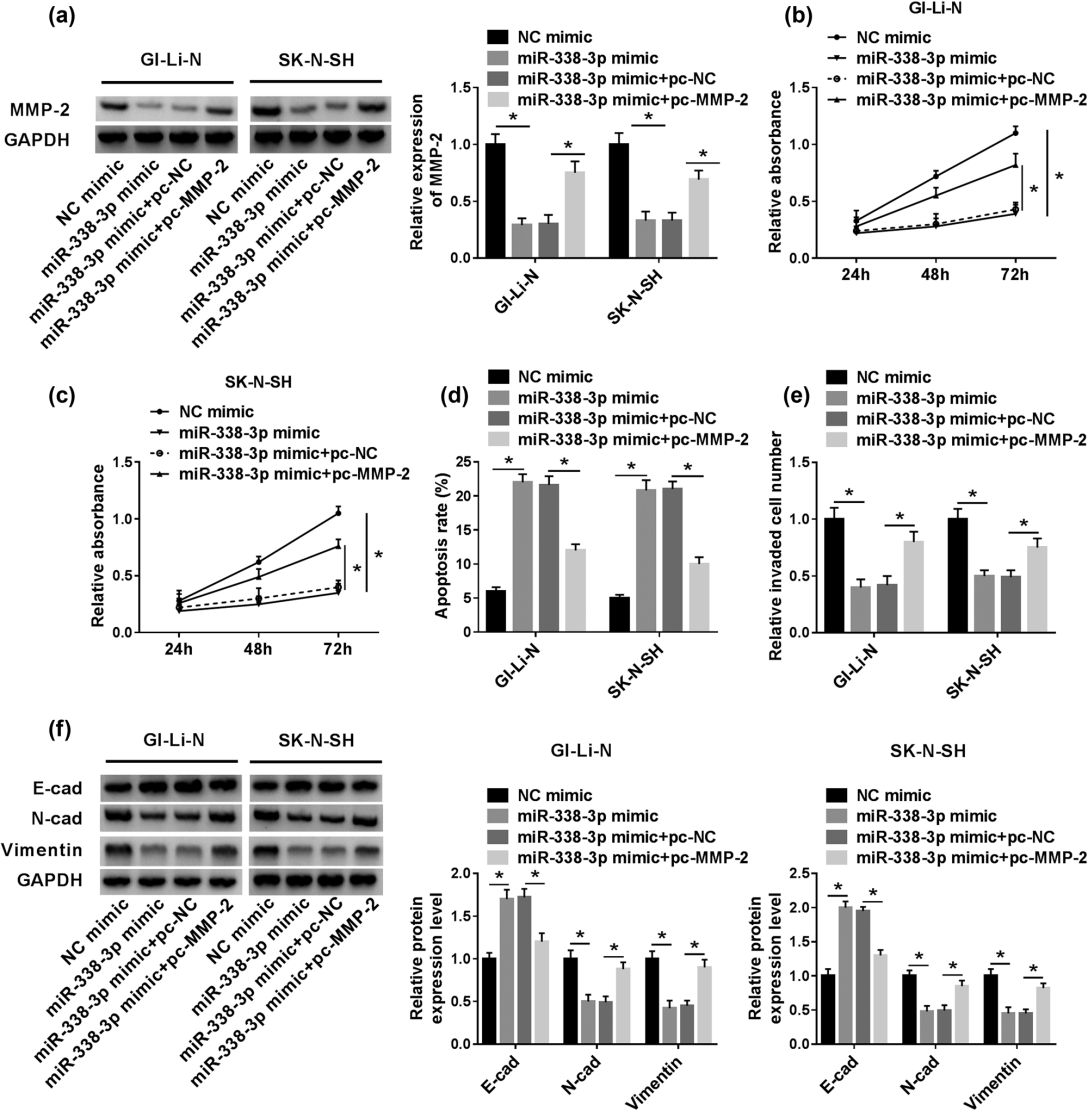
The impeded effect on human neuroblastoma induced by miR-338-3p mimic was restored by pc-MMP-2 *in vitro*. (a) MMP-2 protein expression in miR-338-3p mimic and pc-MMP-2 was assessed by western blot assay. (b and c) The cell proliferation in GI-LI-N and SK-N-SH cells transfected with miR-338-3p or co-transfected with MMP-2 and miR-338-3p was observed by CCK-8 assay at 450 nm. (d) The cell apoptosis rates in miR-338-3p mimic and pc-MMP-2 groups were examined by flow cytometry. (e) The cell invasion numbers in miR-338-3p mimic and pc-MMP-2 groups were measured by transwell assays. (f) EMT progression expressed in miR-338-3p mimic and pc-MMP-2 groups was tested by western blot assays. *P < 0.05.

To further uncover the regulatory mechanism between miR-338-3p and MMP-2 in human neuroblastoma, the recovery experiments were conducted. The results showed that the repressive expression of MMP-2 protein in miR-338-3p mimic groups was specially recuperated by pc-MMP-2 addition (Figure 7a). Meanwhile, the suppression effects of miR-338-3p on cell proliferation and invasion in the two human neuroblastoma cells were partly reversed by MMP-2 overexpression (Figure 7b, c, and e). Subsequently, the boosted cell apoptosis rate in miR-338-3p mimic groups was remarkably overturned by the gain of MMP-2 (Figure 7d). As for EMT progress, the counter-regulated effect of miR-338-3p was evidently relieved by the overexpression of MMP-2 (Figure 7f).

### 3.8 miR-338-3p blocked the PI3K/AKT pathway by sponging MMP-2 in human neuroblastoma cells

**Figure A1:**
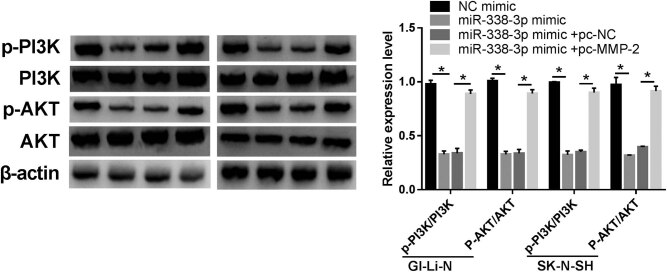
miR-338-3p blocked the PI3K/AKT pathway by sponging MMP-2 in GI-LI-N and SK-N-SH cells. The levels of p-PI3K, PI3K, p-AKT, or AKT in human neuroblastoma cells transfected with NC mimic, miR-338-3p mimic, miR-338-3p mimic + pc-NC, and miR-338-3p mimic + pc-MMP-2 were measured by western blot assays. *P < 0.05.

We further surveyed whether miR-338-3p modulated the PI3K/AKT pathway through sponging MMP-2 in human neuroblastoma cells. We observed that miR-338-3p decreased the ratio of p-PI3K/PI3K and p-AKT/AKT in GI-LI-N and SK-N-SH cells, whereas this impact was reversed after MMP-2 (Figure A1). These results indicated that miR-338-3p sponged MMP-2 to regulate the PI3K/AKT pathway in human neuroblastoma cells.

## 4 Discussion

Neuroblastoma is the most common extracranial solid tumor in infants. Additionally, it can regress spontaneously or grow and acquire resistance to multiple therapeutic approaches [23]. Meanwhile, it has been reported that miRNA research had a great potential impact on the management of neuroblastoma [24]. Subsequently, our study indicated that miR-338-3p indeed had an inhibitive effect on neuroblastoma cell growth, invasion, and EMT process through targeting MMP-2. The results first time revealed the target relationship and regulatory mechanism between miRNA-338-3p and MMP-2 in neuroblastoma.

In addition, our data showed that miR-338-3p was downregulated in neuroblastoma metastasis tissue in total, which was consistent with results obtained in gastric and epithelial ovarian cancer studies [14,15]. Furthermore, miR-338-3p overexpression and the knockdown of MMP-2 could suppress the human neuroblastoma cell proliferation, invasion, and EMT pathway but promote the cell apoptosis. Moreover, the data of upregulated MMP-2 expression also agreed with those reported in glioblastomas and lung cancer [25,26]. Additionally, we observed that MMP-2 was directly targeted by miR-338-3p. It was reported that miRNA-338-3p could directly target PREX2a, MACC1, Rab14, RAB23, IRS2, and Sox4 in ovarian cancer, gastric cancer, breast tumor, prostate cancer, non-small cell lung cancer, glioblastoma, and neuroblastoma [13,14,15,16,17,18]. The target relationship between miRNA-338-3p and MMP-2 provided another evidence for the miRNA regulatory network. Importantly, this study also suggested that MMP-2 could rescue the inhibitive effect of miR-338-3p on neuroblastoma cell progression, cell invasion, and EMT process.

Previous studies presented a close relationship between the upregulation of MMP-2 and the metastatic cancers [27], and the elevated MMP-2 usually predicted a worse prognosis and more aggressive tumor behavior [27,28,29]. It was also reported that MMP-2 controlled the availability of active molecules such as growth factors [30,31], consequently affecting the cell proliferation and cell apoptosis and regulating the EMT process [32,33]. Our data illustrated that miR-338-3p could inhibit the neuroblastoma cell growth, invasion, EMT process, and PI3K/AKT pathway by targeting MMP-2.

The PI3K/Akt pathway plays an essential role in a wide range of biological functions, including metabolism, macromolecular synthesis, cell growth, proliferation, and survival [34]. The activated PI3K/Akt pathway was demonstrated to exert a cancerogenic role in a series of tumors [35]. Liu et al. reported that cholesteatoma epithelial hyperproliferation was associated with the activation of the EGFR/PI3K/Akt/cyclinD1 pathway [36]. In our research, we found that miR-338-3p decreased the ratio of p-PI3K/PI3K and p-AKT/AKT in human neuroblastoma cells, but this effect was reversed after MMP-2. These combined findings indicated that miR-338-3p regulates the PI3K/AKT pathway in human neuroblastoma cells via MMP-2.

In this study, the effects of miRNA-338-3p and MMP-2 on neuroblastoma cell growth and invasion were measured using *in vitro* techniques, and therefore these effects indeed need to be further confirmed by additional studies. However, these results indicate the importance of further investigation of miRNA molecules’ regulatory mechanism in both *in vivo* and clinical experiments.

Overall, the data of this study suggested that miR-338-3p indeed had an inhibitive effect on neuroblastoma cell growth, invasion, and EMT process through targeting MMP-2, which provided a new potential therapeutic target in the treatment of neuroblastoma.
